# Distribution of Natural Radionuclides and ^137^Cs in Urban Soil Samples from the City of Novi Sad, Serbia-Radiological Risk Assessment

**DOI:** 10.3390/toxics11040345

**Published:** 2023-04-05

**Authors:** Marija Janković, Ivana Jelić, Milica Rajačić, Jelena Krneta Nikolić, Ivana Vukanac, Slavko Dimović, Nataša Sarap, Marija Šljivić-Ivanović

**Affiliations:** Radition and Environmental Protection Department, Vinča Institute of Nuclear Sciences, National Institute of the Republic of Serbia, University of Belgrade, 11001 Belgrade, Serbia

**Keywords:** soil, natural radioactivity, gamma spectrometry, gross alpha activity, gross beta activity, health risk

## Abstract

This work presents the natural radioactivity distribution of 21 surface soil samples taken in the city of Novi Sad, Serbia. The analysis for radioactivity was performed using a gas low-level proportional counter for gross alpha and gross beta activity, while the specific activities of radionuclides were determined using HPGe detectors. The gross alpha activity of 20 samples was below the minimum detectable concentration (MDC), while in 1 sample it was 243 Bq kg^−1^; the gross beta activity ranged from the MDC (11 samples) to 566 Bq kg^−1^. The gamma spectrometry measurements showed naturally occurring radionuclides ^226^Ra, ^232^Th, ^40^K, and ^238^U in all investigated samples, with average values (Bq kg^−1^) of 33.9, 36.7, 513.8, and 34.7, respectively. Natural radionuclide ^235^U was detected in 18 samples with activity concentrations in the range of 1.3–4.1 Bq kg^−1^, while in the other 3 samples, the values were below the MDC. The artificial ^137^Cs radionuclide was detected in 90 percent of the samples, with a maximum value of 21 Bq kg^−1^, while the other artificial radionuclides were not detected. Based on the obtained concentrations of natural radionuclides, hazard indexes were estimated, and radiological health risk was assessed. The results present the absorbed gamma dose rate in the air, annual effective dose, radium equivalent activity, external hazard index, and lifetime cancer risk.

## 1. Introduction

Radioactivity in the environment originates from naturally occurring radionuclides, cosmic radiation, and artificial radionuclides. The general population is often exposed to natural levels of radiation that are mainly terrestrial in origin. The term terrestrial radiation considers the radiation coming from the presence of naturally occurring radionuclides belonging to the uranium and thorium series and ^40^K in the soil. The major portion of the radionuclide content, which can be found in the soil, originates from the basic substrate of the Earth’s crust surface. The importance of gross alpha and gross beta activity measurements is primarily due to the eventual radioactive contamination of the environment that results in exposure to humans. An elevated level of beta radiation may be caused by the release of some artificial beta radionuclides, such as ^90^Sr and ^137^Cs [1]. The monitoring of radioactive alpha and beta concentrations is performed by nuclear techniques for environmental samples (solids, liquids, and aerosol filters) [2]. The gross alpha activity in the soil refers to all alpha emitters and depends on the mineralogical composition and geological characteristics of the area. The gross beta activity originates from long-lived radioisotopes such as ^40^K, ^210^Pb, and ^228^Ra. In many studies, the determination of the gross alpha and gross beta activity in soil samples is presented [3,4,5,6,7,8,9,10]. However, industrial processes (such as ore and oil mining, the building industry, etc.) contribute to the so-called technologically enhanced natural radioactivity, which means that the radionuclides present in the raw materials are concentrated in the final product or the waste. Discarding these products in the environment can cause the deterioration of the quality as well as contamination of the soil. All these situations can lead to, as a consequence, the elevated exposure of the public to ionizing radiation [11,12]. In addition to being the main source of continuous radiation exposure to the general population, the soil is an important medium for the transfer of radionuclides to plants and animals, thus representing the indicator of radiological contamination in the environment [13,14,15,16,17]. By knowing the concentration of radionuclides in the soil and analyzing the radiological characterization of the plants growing in that soil, it is possible to estimate the transfer factor of individual radionuclides into roots, stems, leaves, or fruit. This factor depends on the mineralogical composition and chemical and physical properties. The radionuclides in plants originate either from the uptake of radionuclides via the root system or from the atmosphere, where radionuclides are deposited on the above-ground part of the plant [13,14,15,16,17]. The importance of knowing the radiological characteristics of the environment in a certain region is significant in order to create a database for the concentration of radioisotopes, both naturally occurring and artificial, understand their transport in the ecosystem, and model their distribution. Furthermore, knowing the soil radioactivity is important for future radiation hazard assessment, radiation protection, and exploration [18,19]. The concentrations of radionuclides can vary with local geology, altitude, and geomagnetic latitudes. Many activities, including the regular monitoring of both naturally occurring and artificial radionuclide concentrations, are performed in order to assess and control their hazardous effects on the environment since naturally occurring radionuclides as well as ^137^Cs, the remnant from the Chernobyl accident, are of radiological importance to the general population and the environment. These investigations are described in many literature data relating to soil radiological characteristics [13,18,20,21,22,23,24,25,26,27,28,29,30,31,32,33,34,35,36,37,38].

The main objective of this investigation was to conduct a measurement of representative soil samples in the central part of Novi Sad city, determine the radionuclide content and distribution in the soil in the investigated territory, and estimate the potential health risk for the general population as well as the influence on the environment. This study contributes to the knowledge of the natural radioactivity in soil samples in the urban area of Novi Sad city.

## 2. Materials and Methods

### 2.1. Sampling Locations

**Figure 1:**
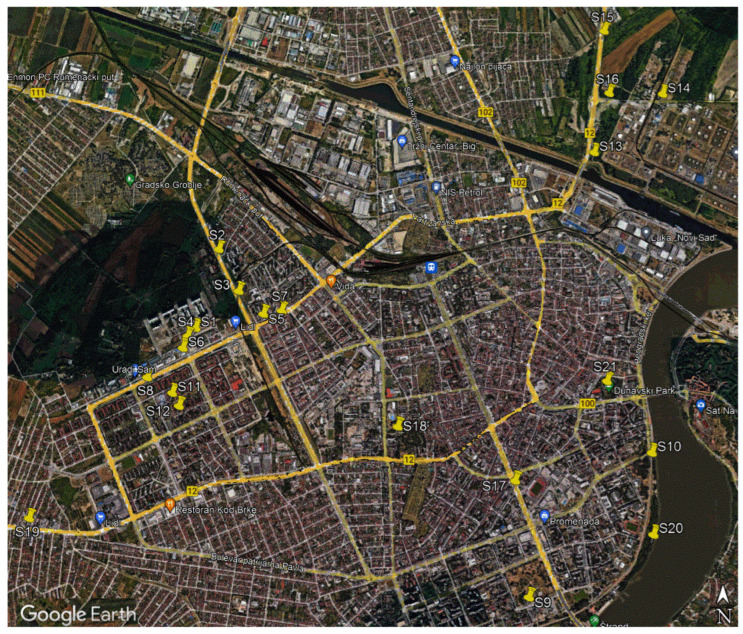
Sampling locations marked as S1–S21 in Novi Sad city.

Soil samples were taken in Novi Sad, the second largest city in the Republic of Serbia, and the capital of Vojvodina (the northern province of Serbia), with a latitude of 45°15′18″ N and a longitude of 19°50′41″ E, an elevation of 80 m, and with 370,000 inhabitants. The urban city area is located on the geological base of alluvial sediments from the Holocene geological period. Alluvial deposits consist of Holocene sandbars and sandy clays as well as Holocene silt and sand [39]. The investigation of urban soil samples was carried out in the central part of the Novi Sad city and the sampling was carried out in 2020. For the purpose of sampling, an adequate number of points in the territory of the city of Novi Sad was chosen in a way that would enable an objective analysis and lead to results that are representative of urban soil samples from different quarters. The sampling was conducted in the vicinity of registered sources of pollution (industry-oil refinery, located 3 km northeast of the city center; four samples marked with S13–S16); in the vicinity of traffic roads in the urban part of the city Avijatičarsko quarter (two samples marked with S2 and S3), Bistrica (three samples marked with S8, S11, and S12), Detelinara (two samples marked with S5 and S7), and Jugovićevo (three samples marked with S1, S4, and S6—located west of the city center); Liman (located in the southeastern part of the city—one sample marked with S9), the center of the city (one sample marked with S17), and one sample taken from the southwest of the city center, road to Veternik settlement (S19); two samples were taken from the city parks in the city center (Danube park marked with S21 and Futog park marked with S18); two samples were taken near the Danube River outside of the influence of the said sources (beside the Danube, sample marked with S10—Sunčani kej (Sunny quay) in the forest, and the sample marked with S20—beside the river. The sampling locations are presented in Figure 1.

### 2.2. Sample Preparation

Samples were taken from the surface layer (0–5 cm). After the sampling, about 1 kg, the samples were labeled and transported to the laboratory for further preparation. The soil samples were cleaned from pebbles, grass, and other debris. After that, the soil was shredded, dried in the oven at a temperature of 105 °C to constant mass, and sifted through a 2 mm sieve. For the gross alpha and gross beta activity measurement, from the total mass of the prepared homogenous sample, about 260 mg was weighed in an aluminum planchet and fixed with alcohol [40]. The final step of preparation for gamma spectrometric measurement included placing the samples into Marinelli beakers and sealing them with beeswax. Then, the prepared samples were left in the laboratory for 30 days in order to achieve a radioactive equilibrium between ^226^Ra and its progenies for gamma spectrometric measurement. The preparation was conducted according to the IAEA Technical Report [41].

### 2.3. Gross Alpha and Gross Beta Activity

For the gross alpha and gross beta activity of soil samples, the gas low-level proportional counter Thermo-Eberline FHT 770T was used. The counting gas is a mixture of 90% Ar and 10% methane. Efficiencies were determined using the certified radioactive calibration standards ^241^Am and ^90^Sr (9031−OL−334/11 and 9031−OL−335/11, respectively, Czech Metrology Institute), traceable to the Bureau International des Poids et Mesures (BIPM). The counting efficiency was 26% for alpha and 35% for beta radiation. The measurement time was 14,400 s, by four independent detectors, simultaneously. The measurement uncertainty was expressed as an expanded measurement uncertainty at a confidence level of 95% (k = 2).

### 2.4. Gamma Spectrometry

Gamma spectrometry of the samples was performed using a High Purity Germanium (HPGe) detector with a relative efficiency of 18% (Gama spectrometer 7229N-7500-1818, Canberra). The calibration of the detector was performed using the certified radioactive standard in Marinelli geometry (1035 SE-40845-17, Czech Metrology Institute, Inspectorate for Ionizing Radiation) which is traceable to BIPM. The radioactive standard contained ^241^Am, ^109^Cd, ^139^Ce, ^57^Co, ^60^Co, ^137^Cs, ^113^Sn, ^85^Sr, ^88^Y, ^51^Cr, and ^210^Pb, with total activity of 80.6 kBq. The measurement duration was 60,000 s and the spectra were analyzed using GENIE 2000 software. The measurement results were expressed as Bq kg^−1^ with a confidence level of 95% (k = 2). The specific activities of the radionuclides were detected via the following gamma energies:

- The activity of ^226^Ra, via the energies of 295 keV, 352 keV, 609 keV, 1120 keV, and 1764 keV from its progenies ^214^Pb and ^214^Bi;
- The activity of ^232^Th via the energies of 338 keV and 911 keV from its progeny ^228^Ac;
- The activity of ^40^K via the energy of 1460 keV;
- The activity of ^235^U via the energy of 143 keV and 186 keV, which was corrected for the contribution from ^226^Ra;
- The activity of ^238^U via the energy of 63 keV from its progeny ^234^Th or via 1000 keV from its progeny ^234m^Pa;
- The activity of artificial radionuclide ^137^Cs via the energy of 661 keV.

### 2.5. Hazard Indexes Calculation

Based on the obtained activity concentrations of naturally occurring radionuclides ^226^Ra, ^232^Th, and ^40^K in investigated soil samples, assuming that the other radionuclides can be neglected because their contribution to the total dose from an environmental background is small, the outdoor absorbed gamma dose rate in the air and the radium equivalent activity and external hazard index can be determined. Radiological risks also can be determined by assessing the annual effective dose and lifetime cancer risk (LTCR).

#### 2.5.1. Outdoor Absorbed Gamma Dose Rate in Air

The outdoor absorbed gamma dose rate in the air, in nGy h^−1^, can be calculated using the following equation [42]:

(1) $$D=0.462\times A_{Ra}+0.604\times A_{Th}+0.417\times A_{K}.$$

where *D* is the dose rate in the air at 1 m above the ground surface, *A_Ra_*, *A_Th_*, and *A_K_* are the activity concentrations of ^226^Ra, ^232^Th, and ^40^K (Bq kg^−1^), respectively, while 0.462, 0.604, and 0.0417 are the conversion factors for these radionuclides.

#### 2.5.2. Annual Effective Dose Rate

The annual effective dose rate *D_E_* in mSv, taking into account the coefficient of transferring from absorbed dose in the air to effective dose (0.7 Sv Gy^−1^) and occupation factor for the outdoors (20%), can be calculated by the following equation [42]:

(2) $$D_{E}=0.7\times 0.2\times 8760\times D$$

where 8760(h) is the outdoor annual exposure time (365 × 24 h).

#### 2.5.3. Radium Equivalent Activity

According to the fact that the distribution of ^226^Ra, ^232^Th, and ^40^K in soil is not uniform, the radium equivalent activity was determined through the following relation:

(3) $$Ra_{eq}=A_{Ra}+1.43\times A_{Th}+0.077\times A_{K}$$

While defining *Ra_eq_* activity, it has been assumed that 1 Bq kg^−1^ of ^226^Ra or 1.43 Bq kg^−1^ of ^232^Th or 0.077 Bq kg^−1^ of ^40^K produce an equal gamma dose rate [43].

#### 2.5.4. External Hazard Index

The external hazard index (*H_ex_*) represents the widely used value that reflects the risk of external exposure to ionizing radiation. It is defined in [42] and is calculated according to the following equation:

(4) $$H_{ex}=A_{Ra}/370+A_{Th}/259+A_{K}/4810$$

where *A_Ra_*, *A_Th_*, and *A_K_* are the activity concentration of ^226^Ra, ^232^Th, and ^40^K respectively (in Bq kg^−1^).

#### 2.5.5. Lifetime Cancer Risk

The lifetime cancer risk can be calculated using the following equation:

(5) $$LTCR=D_{E}\times DL\times RFSE$$

where *DL* is the average duration of a lifetime (estimated 70 y) and *RFSE* (Sv^−1^) is the risk factor for stochastic effects and is defined to be 0.05 Sv^−1^ for the common population. This index expresses the probability of developing cancer over a lifetime at a given exposure level [43,44].

## 3. Results and Discussion

### 3.1. Gross Alpha and Gross Beta Activity

The obtained results for the gross alpha and gross beta activity in the 21 soil samples are given in Table 1. All samples, except one, have a gross alpha activity below the minimum detectable concentration. The minimum detectable concentrations for gross alpha activity were in the range of <156 to <306 Bq kg^−1^. The gross beta activity ranged from MDC to 566 Bq kg^−1^. The MDC for gross beta activity was in the range of <158 to <198 Bq kg^−1^. The obtained gross beta activity is mainly due to the presence of ^40^K but also ^137^Cs, while the gross alpha activity in the samples originates from the decay chains of ^238^U and ^232^Th. The counting system for gross alpha and gross beta activity determination includes three manually operated sample slides, each with two measuring positions. This enables the simultaneous determination of gross alpha and gross beta activity in alpha mode, beta mode, or alpha/beta mode. For all detectors, the efficiencies for alpha and beta radiation must be determined and these values can differ slightly. The background for each position was obtained by counting clean empty planchets. Bearing in mind that efficiency and background enter into the equation for gross alpha/beta determination, the calculated MDC differs from position to position where the samples were measured. To avoid the influence of self-absorption, the quantity of residue in the planchet must be calculated for a certain mass thickness. However, problems with self-absorption for the determination of gross alpha activity in solid deposits with the gas proportional counter can be present in thick sources. This factor can be dominant in gross alpha activity determination when the range of the emitted particles is less than the thickness of the source, but this does not apply when determining gross beta activity [45]. The maximum calculated quantity in the planchet for gross alpha activity is 130 mg, for which self-absorption will not occur; the value for gross beta is 260 mg. Since the determination of gross alpha and gross beta activity is a screening method, specific analyses for radionuclides were performed using gamma spectrometry.

### 3.2. Gamma Spectrometry Measurement Results

Results of the gamma spectrometry measurement of the 21 soil samples with statistical data are presented in Table 2. All the analyzed samples show the presence of naturally occurring radionuclides ^226^Ra, ^232^Th, ^40^K, and ^238^U. The activity concentrations (Bq kg^−1^) were in the range of 15–51 for ^226^Ra, with an average value of 33.9, 18–62 for ^232^Th, with an average value of 36.7, 210–620 for ^40^K, with an average value of 513.8, and 18–85 for ^238^U, with average value 34.7. The minimum values for ^226^Ra ^232^Th, ^40^K, ^238^U, and ^235^U were obtained in soil sample S12 at the location Bistrica (near the traffic roads in the urban part of the city). Interestingly, the second sample taken in the vicinity, S11, shows the highest value for ^40^K, ^238^U, and ^235^U. The activity concentration of ^226^Ra has the highest value for location S20 (Sunčani kej (Sunny quay) near Danube), while ^232^Th has the highest value for one location near the entrance to the oil refinery (S14). There was no significant difference between the activity concentrations of radionuclides taken from different quarters in the urban part of the city. Locations in the Bistrica (S8, S11, S12), Detelinara (S5, S7), Jugovićevo (S1, S4, S6), and Avijatičarsko quarters (S2, S3) are close to each other on the west side of the city. There was also no significant difference between the obtained results for these locations and the results for the Liman (S9) quarter located in the southeastern part of the city, the center of the city (S17), and a sample taken from the southwest of the city center, the road to Veternik settlement (S19). Soil samples taken from the city parks S18 (Futog park) and S21 (Danube park) in the central part of the city have similar values for the activity concentrations of radionuclides and similar values as the samples mentioned above. The results obtained for soil samples from locations beside the Danube (S10-Sunčani kej (Sunny quay) in the forest) and S20 (beside the river) show some differences; ^226^Ra, ^232^Th, ^238^U, and ^235^U have a higher value for sample S20. Samples taken in the vicinity of the oil refinery, S13–S16, have activity concentrations of radionuclides in the same order of magnitude as the other investigated samples.

Natural radionuclide ^235^U was detected in 18 samples with activity concentrations in the range of 1.3–4.1 Bq kg^−1^, while in the other 3 samples, the values are below the MDC.

In general, the activity concentration of ^40^K in soil is one order of magnitude higher than the concentrations of ^226^Ra, ^232^Th, and ^238^U. The mean values for ^226^Ra and ^238^U obtained in this investigation for soil samples, 33.9 Bq kg^−1^ and 34.7 Bq kg^−1^, respectively, are below the worldwide average values for these radionuclides, 35 Bq kg^−1^, while the mean values for ^232^Th (36.7 Bq kg^−1^) and ^40^K (513.8 Bq kg^−1^) are higher than the world average values of 30 and 400 Bq kg^−1^, respectively [42].

Artificial radionuclide ^137^Cs was detected in 90 percent of the samples, ranging from 1.4 to 21 Bq kg^−1^, while the other artificial radionuclides were not detected. The highest value was obtained for location S20 (Sunčani kej (Sunny quay) near Danube). The presence of this radionuclide in the surface soil is a consequence of the Chernobyl accident and atmospheric nuclear tests up to the 1980s. ^137^Cs was emitted into the atmosphere along with the other artificial radionuclides. Due to his long half-life (30 y), this radioisotope is still present in the environment [46]. In the case of undisturbed or uncultivated soil, the concentration of ^137^Cs is generally higher in the surface profile (0–10 cm depth) and the migration is slow. The disturbed soil redistribution of this radionuclide is associated with mechanical mixing [23,47]. In [48], the radioactivity of soil samples in Vojvodina (the northern province of Serbia), including Novi Sad city, in 2001 and 2010 was examined. The study noted a slight decrease in ^137^Cs in the surface soil after 2001. We can notice the same trend in this work as well and this is very important because ^137^Cs has a chemical analogy with potassium and represents one of the most hazardous artificial radionuclides. Radioisotopes of ^137^Cs in the soil behave the same as stable cesium. When it enters in soil, by deposition from the atmosphere, it migrates slowly because of adsorption on the clay fraction and organic matter [49]; organic matter accumulates ^137^Cs and reduces its mobility [49]. The vertical migration of this radioisotope in the soil is estimated to be 0.1–1 cm per year [49]. Before the accident in Chernobyl in 1986, the concentration of this radionuclide in the soil in Serbia was below 5 Bq kg^−1^ [50]. In 1991, in limestone soil samples from Tara Mountain in Serbia, the activity concentration of this radioisotope was below 100 Bq kg^−1^. In 1997, in soil samples from Šara Mountain in Serbia, the activity concentration of this radioisotope was below 100 Bq kg^−1^, while in soil from Stara Mountain in 2000, its activity was 50 Bq kg^−1^ [50]. Until 1996, ^137^Cs remained on the surface layer of soil, except on riverbanks, due to the washout effect [50]. In this work, as mentioned above, the highest value was obtained for the location near the river. In Belgrade, 25 years after Chernobyl, in undisturbed soil samples, the mean activity of ^137^Cs was 29.9 Bq kg^−1^ [51]. From 1987 to 1988 the concentration in Belgrade ranged from 975 to 2925 Bq kg^−1^, which is almost a hundred times more than in 2011 [51]. In May 1986, the activity of ^137^Cs was 1.6 kBq kg^−1^ in Belgrade soil [51].

In order to analyze the degree of correlation between radionuclides, Pearson’s linear correlation analysis was applied. The results are presented in Table 3. A significant positive correlation exists between natural radionuclides which have a common source (correlated among themselves) (0.73–0.75). Between ^238^U and ^226^Ra, ^232^Th and ^40^K, a relatively weak but still significant correlation exists caused by the high mobility of ^238^U [52]. The absence of a correlation between artificial radionuclide ^137^Cs and members of the uranium decay series and ^40^K arises from their different sources. A strong correlation exists between ^235^U and ^238^U, ^232^Th, and ^40^K and a positive correlation exists between ^235^U and ^226^Ra.

In most natural systems, the activity ratio parent/parent (^238^U and ^232^Th series) is relatively constant [12]. If a secular equilibrium exists between parents of the ^238^U and ^232^Th series with their progenies, the activity ratio ^238^U/^226^Ra will be approximately 1. The activity ratio between members of the ^238^U and ^232^Th decay chain (^232^Th/^226^Ra) in most environmental samples is about 1.1 [23,52]. Table 4 presents the ^238^U/^226^Ra and ^232^Th/^226^Ra activity ratios. The mean value for the ^238^U/^226^Ra activity ratio is 1.02. Due to the higher activity concentrations of ^228^U than ^226^Ra at nine sampling locations, the ratio is above 1, and these two radionuclides are in disequilibrium due to the different mobility of these radioisotopes [23,52]. ^226^Ra has very low mobility compared to ^238^U [23,52]. The calculated ^232^Th/^226^Ra activity ratio was in the range of 0.86–1.44, with a mean value of 1.09 which is in accordance with the ratio of 1.1 in environmental samples. The ^235^U/^238^U ratio indicates the natural origin of the two uranium isotopes.

**Figure 2:**
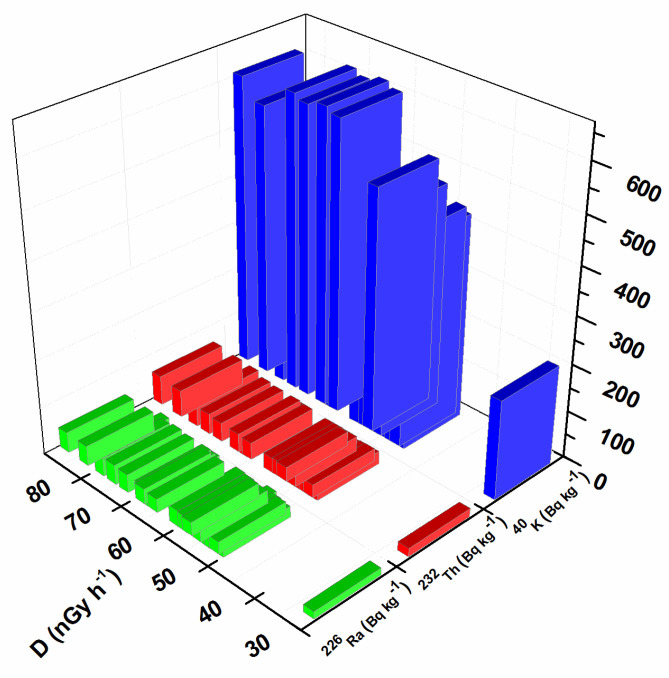
Outdoor absorbed gamma dose rate.

**Figure 3:**
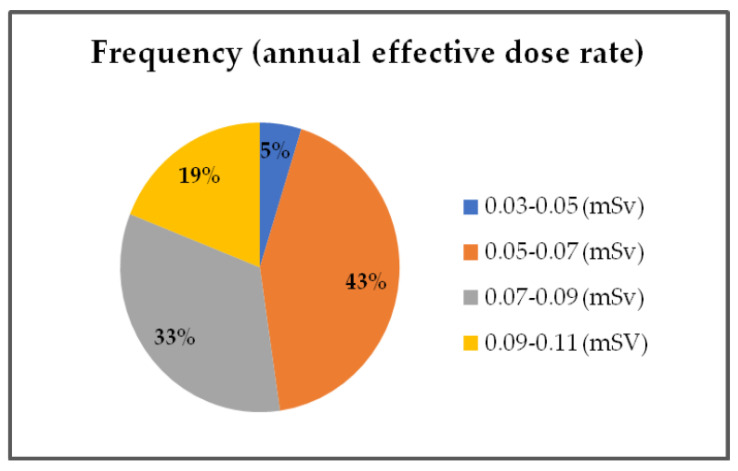
Annual effective dose rate.

**Figure 4:**
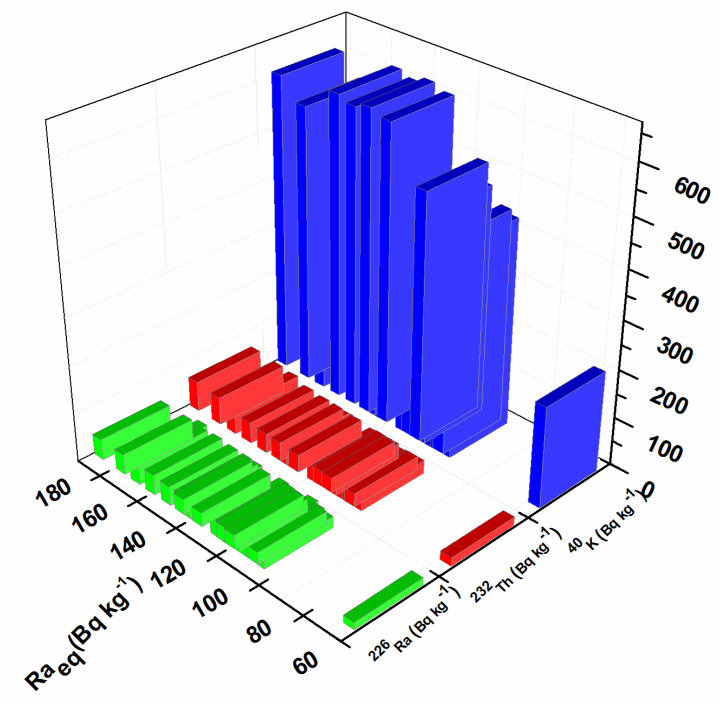
Radium equivalent activity.

**Figure 5:**
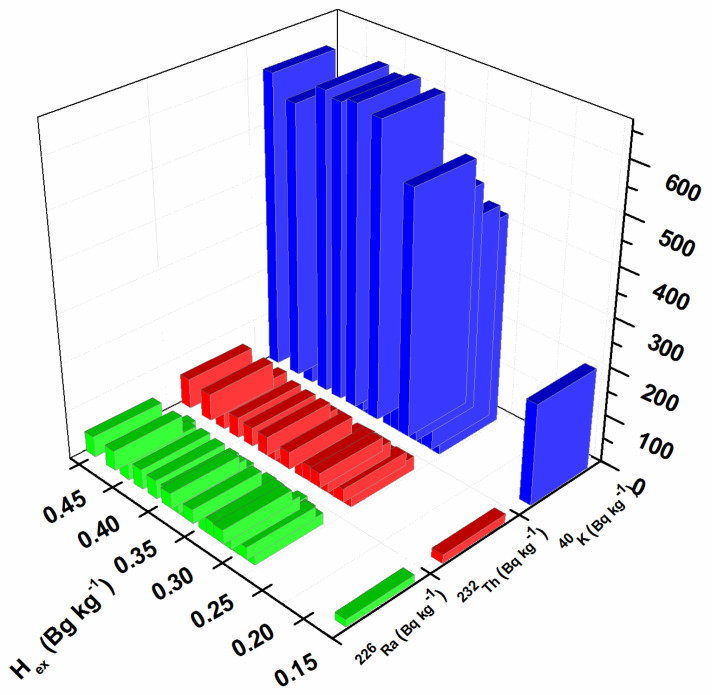
Hazard index.

In order to assess the impact of natural radioactivity from the investigated soil samples on the population, the outdoor gamma absorbed dose rate, annual effective dose, radium equivalent activity, hazard index, and lifetime cancer risk were evaluated. External exposure outdoors originates from the terrestrial radionuclides present in the soil and depends on the types of rocks (granite or sedimentary) [42]. The results obtained for the outdoor gamma absorbed dose in the air, calculated using Equation (1), are presented in Figure 2. The calculated values were in the range of 27–83 nGy h^−1^. The maximum value for the gamma absorbed dose rate was calculated for sample S14 as 83 nGy h^−1^ (near the oil refinery), which is in correspondence with the higher values of ^226^Ra, ^232^Th, and ^40^K. The minimum value was calculated for sample S12, in the amount of 27 nGy h^−1^ (Bistrica quarter) which is in agreement with the lowest values of ^226^Ra, ^232^Th, and ^40^K. The mean value of the outdoor gamma absorbed dose in the air for all investigated locations was 59 nGy h^−1^, which is in accordance with the world mean value of 59 nGy h^−1^ [42]. The frequency of the annual effective dose is presented in Figure 3. The highest percent of the calculated annual effective dose is around the world average value (0.07 mSv): 43% have values between 0.05 and 0.07 mSv and 33% have values between 0.07 and 0.09%. Only 5% of calculated values are below 0.05 mSv, and 19% have values above 0.09 mSv. The highest annual effective dose was 0.101 mSv, which is in accordance with the highest obtained outdoor gamma absorbed dose rate. The smallest annual effective dose was 0.033 mSv, which is in accordance with the smallest obtained outdoor gamma absorbed dose rate. The obtained results for radium equivalent activity are presented in Figure 4. The calculated values were in the range of 57 to 179 Bq kg^−1^. These values for all investigated soil samples are lower than the safety limit of 370 Bg kg^−1^. The distribution of the hazard index is given in Figure 5. In order to keep the annual effective dose below the limit of 1.5 mGy y^−1^, the hazard index *H_ex_* should not exceed 1. As can be seen from Figure 5, all values are below 1 and it can be concluded that the radiation risk from gamma radiation is negligible. The calculated lifetime cancer risk ranged from 1.16 to 3.56 × 10^−4^ with an average value of 2.55 × 10^−4^. The mean value for lifetime cancer risk is lower than the world’s average value, 2.90 × 10^−4^ [24].

**Figure 6:**
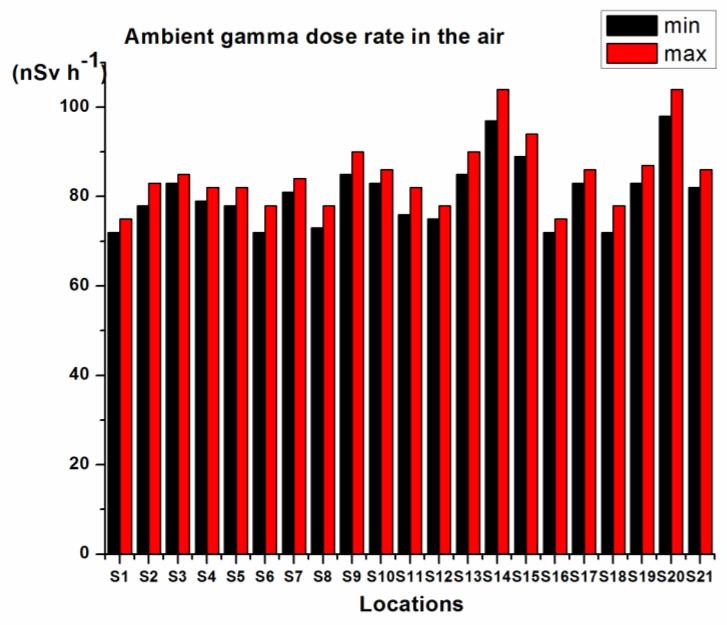
Ambient gamma dose rate in the air.

Figure 6 presents the measured ambient gamma dose rate in the air at 1 m above the ground surface at the locations where soil samples were collected. The measurements were performed using an Automess Scintillator Probe 6150 Adb. The figure presents minimum and maximum values for each location. The highest value was measured at location S14 (97–104 nSv h^−1^) and location S20 (98–104 nSv h^−1^), which is in good agreement with the higher values of detected concentrations of radionuclides.

Table 5 presents the comparison of gamma-emitted radionuclides in soil samples in the literature. The comparison is performed with soil samples from different countries in the world, countries around Serbia, as well as from different cities or regions in Serbia. Results for the activity concentrations of radionuclides in soil samples from Spain [52] and Brazil [24] are in good agreement with the results obtained in this work. In China [25], ^40^K has a higher value than the concentrations of ^40^K obtained in this work; on the other hand, the ^232^Th in soil samples from India [26] has a higher value than the concentrations of ^232^Th obtained in this work. Investigations of soil samples from city parks in Belgrade [23] also show similar results as soil samples in the city of Novi Sad.

The comparative analysis of the results obtained in Serbia (Novi Sad city) with the results obtained for soil samples from surrounding counties (North Macedonia, Republic of Srpska, Croatia, Montenegro, and Slovenia [27,28,29,30,31]) leads to the conclusion that the activity concentrations of the detected radionuclides are of the same order of magnitude and comparable.

The analysis of soil samples in other cities in Serbia (Niš, Čačak, Kragujevac [32,33,34,35]) shows agreement with the results presented in this work. Research conducted at the Serbian Mountains Zlatibor (elevation 1030 m) and Kopaonik (elevation 2017 m) show higher values of ^137^Cs than the values obtained in this work and the other cities in Serbia. At Kopaonik, the activity concentrations of ^226^Ra, ^232^Th, and ^40^K are also higher than the values obtained for soil samples from Novi Sad city. At Zlatibor Mountain [36,37], the values of ^232^Th, ^238^U, and ^40^K are smaller than the values obtained in this work. The results presented for the activity concentrations of radionuclides in Belgrade soil samples and the samples from Belgrade municipalities Lazarevac and Obrenovac [53] show very similar results to this work.

In addition, there was no significant difference between the radioactivity content in uncultivated soil (this work) and cultivated agricultural soil systems in the Novi Sad city [47] and Belgrade [13].

The content of radionuclides in the soil samples analyzed in this work can also be compared to soil samples collected in the vicinity of coal-fired power plants in Serbia. The deposition of fly ash particles, as a by-product in the combustion process of coal in power plants, can cause an environmental hazard due to the enrichment of radionuclides in the by-products after combustion. This process can have an effect on the soil in the surrounding areas. The results obtained for soil samples in the vicinity of four coal-fired power plants in Serbia show similar results to the soil samples investigated in this work [38].

## 4. Conclusions

Surface soil samples from the urban part of the city of Novi Sad, Serbia, collected at 21 locations, were analyzed for gross alpha and gross beta activities using a gas low-level proportional counter. In order to determine the specific activities of natural radionuclides, as well as the artificial radionuclide ^137^Cs, soil samples were analyzed by gamma spectrometry using HPGe detectors. The determination of the gross alpha and gross beta activity represents a screening method that refers to the gross alpha or gross beta emitted radionuclides. However, determining the content of radionuclides qualitatively and quantitatively requires a more sophisticated method, such as gamma spectrometry. Naturally occurring radionuclides ^226^Ra, ^232^Th, ^40^K, and ^238^U were detected in all analyzed samples with average values (Bq kg^−1^) of 33.9, 36.7, 513.8, and 34.7, respectively. The natural radionuclide ^235^U was detected in 18 samples with activity concentrations in the range of 1.3–4.1 Bq kg^−1^, while in the other 3 samples, the values are below the MDC. Artificial radionuclide ^137^Cs were detected in 19 samples in the range of 1.4–21 Bq kg^−1^, mainly as a consequence of the Chernobyl accident.

A good correlation was observed between natural radionuclides, which have a common source, and a very strong correlation was observed between ^235^U and ^226^Ra, ^232^Th, and ^40^K.

The ^238^U/^226^Ra and ^232^Th/^226^Ra activity ratios, with mean values of 1.02 and 1.09, show the different mobility of these radionuclides in soil.

The mean calculated gamma absorbed dose rate in the air (59 nGy h^−1^) is in good agreement with the world average value (59 nGy h^−1^). In addition, the largest number of calculated annual effective doses is around the world’s mean value (0.07 mSv). The radium equivalent activity and external hazard index are below the safety limit and the estimated lifetime cancer risk has a mean value of 2.55 × 10^−4^, which is lower than the world average.

By comparing the results obtained in this work with the results available in the literature for other soil types, there are no significant differences in radioactivity content. Based on the obtained results of activity concentrations and calculated hazard indexes, the soil samples from the investigated region do not pose a risk of high radiation exposure to the population and can be used for any purpose.

## Figures and Tables

**Table 1:** Gross alpha (Bq kg^−1^) and gross beta activity (Bq kg^−1^) in the 21 investigated soil samples.

No.	Gross Alpha Activity	Gross Beta Activity
S1	<253	<191
S2	<156	460 ± 53
S3	<221	400 ± 53
S4	<306	<198
S5	<214	210 ± 49
S6	<226	<174
S7	<221	<158
S8	<248	<172
S9	<228	<179
S10	<214	285 ± 52
S11	<247	<169
S12	<222	252 ± 51
S13	<162	427 ± 50
S14	<168	512 ± 54
S15	243 ± 50	566 ± 54
S16	<226	<174
S17	<221	<166
S18	<227	<180
S19	<209	195 ± 51
S20	<234	360 ± 58
S21	<233	<179

**Table 2:** Activity concentrations of radionuclides in the investigated soil samples (Bq kg^−1^).

No.	^226^Ra	^232^Th	^40^K	^238^U	^235^U	^137^Cs
S1	29 ± 2	30 ± 3	510 ± 40	28 ± 7	1.6 ± 0.3	<0.03
S2	29 ± 2	36 ± 3	610 ± 40	20 ± 5	<0.5	6.0 ± 0.5
S3	32 ± 2	39 ± 3	620 ± 40	22 ± 5	<0.5	3.9 ± 0.4
S4	32 ± 2	33 ± 3	470 ± 30	27 ± 4	1.7 ± 0.2	1.4 ± 0.3
S5	30 ± 2	32 ± 3	470 ± 30	32 ± 7	1.7 ± 0.3	<0.2
S6	27 ± 1	32 ± 3	470 ± 30	25 ± 4	<0.4	2.5 ± 0.3
S7	38 ± 3	39 ± 5	550 ± 40	39 ± 9	2.0 ± 0.3	5.5 ± 0.9
S8	26 ± 1	28 ± 3	440 ± 30	24 ± 5	1.7 ± 0.2	5.7 ± 0.5
S9	39 ± 2	40 ± 3	610 ± 40	39 ± 6	2.4 ± 0.2	7.5 ± 0.6
S10	36 ± 2	36 ± 4	590 ± 40	33 ± 6	2.2 ± 0.3	8.7 ± 0.9
S11	42 ± 3	43 ± 5	620 ± 40	85 ± 10	4.1 ± 0.5	4.6 ± 0.8
S12	15 ± 1	18 ± 2	210 ± 10	18 ± 3	1.3 ± 0.2	1.5 ± 0.2
S13	42 ± 2	57 ± 2	570 ± 40	46 ± 6	3.4 ± 0.3	2.2 ± 0.4
S14	43 ± 2	62 ± 5	610 ± 40	49 ± 6	3.2 ± 0.3	4.2 ± 0.5
S15	39 ± 2	54 ± 4	560 ± 40	45 ± 6	3.1 ± 0.3	1.6 ± 0.3
S16	29 ± 2	27 ± 2	430 ± 30	31 ± 4	1.4 ± 0.1	5.2 ± 0.4
S17	37 ± 3	25 ± 2	410 ± 30	26 ± 4	1.3 ± 0.1	4.4 ± 0.4
S18	24 ± 2	28 ± 3	430 ± 30	33 ± 10	1.6 ± 0.2	4.9 ± 0.5
S19	37 ± 2	36 ± 3	550 ± 30	32 ± 5	1.9 ± 0.2	2.7 ± 0.4
S20	51 ± 2	44 ± 3	580 ± 40	41 ± 6	2.4 ± 0.2	21 ± 1
S21	35 ± 2	32 ± 3	480 ± 30	33 ± 4	2.3 ± 0.2	2.3 ± 0.2
Mean	33.9	36.7	513.8	34.7	1.9	4.6
St. deviation	7.9	10.8	99.5	14.4	0.9	4.4
Minimum	15	18	210	18	0.4	0.03
Median	35	36	550	32	1.7	4.2
Maximum	51	62	620	85	4.1	21

**Table 3:** Pearson correlation coefficients between the radionuclides in the soil samples.

Variable	^238^U	^226^Ra	^232^Th	^40^K	^235^U	^137^Cs
^238^U	1					
^226^Ra	0.63	1				
^232^Th	0.59	0.75	1			
^40^K	0.49	0.73	0.74	1		
^235^U	0.90	0.67	0.84	0.72	1	
^137^Cs	0.11	0.49	0.10	0.33	0.02	1

**Table 4:** Activity ratios.

No.	^238^U/^226^Ra	^232^Th/^226^Ra
S1	0.97	1.03
S2	0.69	1.24
S3	0.69	1.22
S4	0.84	1.03
S5	1.07	1.07
S6	0.93	1.19
S7	1.03	1.03
S8	0.92	1.08
S9	1	1.03
S10	0.92	1
S11	2.02	1.02
S12	1.20	1.20
S13	1.10	1.36
S14	1.14	1.44
S15	1.15	1.38
S16	1.07	0.93
S17	0.70	0.68
S18	1.38	1.17
S19	0.86	0.97
S20	0.80	0.86
S21	0.94	0.91
Mean	1.02	1.09

**Table 5:** Comparison of the content of radionuclides in the soil samples from different countries and cities or regions in Serbia.

Country	^226^Ra	^232^Th	^40^K	^238^U	^235^U	^137^Cs	Reference
Spain	27	35	590	40		31	[52]
China	25	29	923	26		5.6	[25]
Brazil	28		630				[24]
India	53	203	479				[26]
North Macedonia	38.8	43.7	546			41.5	[27]
Republic of Srpska	47	41	536	64	3.4	26	[28]
Croatia	69	60	418	61	2.6		[29]
Montenegro	28.6	43.1	620.8			55	[30]
Slovenia	63	77	800	34			[31]
Serbia (Niš) southeast	21	26	414			4.7	[32]
Serbia west	33.2	49.1	379	60.4		36.4	[33]
Serbia (Kragujevac) central	33.5	50.3	425.8			40.2	[34]
Serbia (Čačak) central	26.8	35.1	433.8	47.1		42.8	[35]
Serbia (Zlatibor) southwest		17.9	142	27.1		232	[36]
Serbia (Kopaonik) southwest	80	77	725			4.2–142	[37]
Serbia Belgrade	27.5–47	31–49	510–620	32–61	1.4–2.5	3–47	[53]
Serbia Belgrade city parks	33–50	28–50	424–576	14–46	1.2–3.4	0.7–35.8	[23]
Serbia Lazarevac	25.1–58	45.9–63	470–586	45–54	1.8–3.2	28.6–65	[53]
Serbia Obrenovac	33.1–41	51	580–660	39–50	2.4–2.7	15–22.3	[53]
This work	33.9	36.7	513.8	34.7	1.9	4.6	

## Data Availability

Not applicable.
